# Correlation between saliva and serum concentrations of estradiol in women undergoing ovarian hyperstimulation with gonadotropins for IVF/ICSI

**Published:** 2017-06

**Authors:** C Dielen, T Fiers, S Somers, E Deschepper, J Gerris

**Affiliations:** UZ Gent, Dept of Reproductive medicine, De Pintelaan 185, 9000 Gent, Belgium; UZ Gent, Clinical Pathology dept., De Pintelaan 185, 9000 Gent, Belgium; Biostatistical Unit, Ugent. De Pintelaan 185, 9000 Gent, Belgium

**Keywords:** ovarian stimulation, saliva, serum, estradiol, measurement, correlation

## Abstract

**Aim of the study:**

To examine saliva- and serum concentrations correlation of estradiol (E2) in women undergoing ovarian hyperstimulation for IVF/ICSI. Saliva measurements could simplify stimulation follow up. A ‘home’ test for E2 could be useful.

**Methods:**

Prospective interventional academic monocentric study at the Centre for Reproductive Medicine of the University Hospital of Ghent, Belgium. Between November 2014 and August 2015 thirty-one patients were included after random selection (inclusion criteria: < 41 years of age, any rank of IVF/ICSI cycle, serum anti- Müllerian hormone concentration ≥ 1 μg/L, treatment completely at the University Hospital.) Measurements took place using immunoassay serum measurements. Estradiol was determined in saliva and serum by LC-MS/MS. At every control, E2 was measured in saliva and serum. Equilibrium analysis on a part of the serum samples took place. Statistic method used is a linear Mixed- Effects model (MIXED) in SPSS.

**Results:**

Statistical analysis shows a strong linear relation between serum and salivary E2, (R2 of 0.75). E2 in equilibrium dialysis and E2 in serum were also strong correlated (R2 of 0.85).

**Conclusions:**

Strong correlation between serum and salivary E2 concentrations was found. Equilibrium dialysis showed good correlation with salivary E2. Saliva can be a good surrogate for free E2 in women undergoing ovarian hyperstimulation. This may create an opportunity to develop a point of care test for measuring E2, in purpose to simplify screening for OHSS risk.

## Introduction

Close monitoring of stimulation cycles of patients undergoing assisted reproductive treatment (ART), in vitro fertilization (IVF)/ intracytoplasmatic sperm injection (ICSI), is needed in order to follow the number and size of developing follicles, to adapt if needed the dose of gonadotropins and for timing of hCG administration, prior to oocyte retrieval. Monitoring is also used in the prevention of ovarian hyperstimulation syndrome (OHSS), which is a serious and potentially life – threatening condition. The incidence of OHSS is estimated to range from 0.2% to 2.7% of all assisted reproductive cycles, including intra-uterine insemination. (Smitz et al., 1990; MacDougall et al., 1992; Navot et al., 1992; Roest et al., 1996; Nygren and Andersen , 2002). Serial transvaginal ultrasound examinations (TVUS) are used for monitoring. Although blood sampling is controversial in ART follow up (ref. Cochrane) some fertility units measure serum estradiol (E2) concentrations to obtain additional information about the ovarian response and the potential risk of hyperstimulation. Progesterone (P4) measurements may also be performed. Close monitoring however has some downsides for the patient, the care providers and for society. Patients need to visit a care provider (gynaecologist, IVF physician, nurse or midwife), which implies transportation and productivity loss. It stresses patients, partners, care providers and the environment and it adds to the costs of treatment. Patients living at longer distances have a more difficult or even no access to treatment.

Monitoring patients at a distance, by teaching them to make TVUS at home and send the images to their care provider, who interprets them, has been previously explored (Gerris et al., 2009, 2010). Gerris et al. (2014) published a prospective randomized controlled trial about Self- Operated Endovaginal Telemonitoring (SOET) at home versus traditional monitoring of ovarian stimulation in ART. They found similar conception rates, on-going pregnancy rates, numbers of metaphase II (MII) oocytes retrieved and numbers of top quality embryos, indicating non-inferiority of SOET. Patient reported outcomes and health economic analyses were in favour of SOET.

The question remains whether ovarian hyper- stimulation needs to be followed by TVUS only or by TVUS and serum E2 measurements. Serum E2 has been demonstrated to be partly a predictor for OHSS and patients with high E2 levels on the day of the ovulation trigger are at increased risk of OHSS (Kummer et al., 2011). Lee et al. (2008) investigated the value of E2 and anti-Müllerian hormone in the prediction of OHSS and concluded that serum E2 level on the day of hCG administration was a significant predictor of OHSS. Kummer et al. (2011) found that an E2 level of ≥ 4,000 pg/mL on the day of the GnRH-agonist trigger is an important predictor of OHSS development in high-risk patients. In the study of Gera et al. (2010) the overall incidence of OHSS for those who had an estradiol level >2500 pg/mL was 20.2% (38 out of 188). D’Angelo et al. (2004) showed a serum E2 level of 12,315 pmol/L and higher (3,354 pg/mL) on day 11 of ovarian stimulation yield a sensitivity and specificity of 85% for the detection of women at risk for OHSS. Aboulghar et al. (2003) concluded that, irrespective of the debatable role of estrogens in the pathogenesis of OHSS, there is a general agreement that E2 is an important marker to detect the majority of patients at risk for OHSS.

However, a Cochrane review of randomized controlled trials found no good evidence suggesting that combined monitoring by TVUS and serum E2 is more efficacious than monitoring by TVUS alone, both from the point of view of clinical pregnancy rates and the incidence of OHSS (Kwan et al., 2014). The number of oocytes retrieved was similar for both monitoring protocols. However, these results should be interpreted with caution because the overall quality of the evidence was low. A combined monitoring protocol, TVUS and serum E2, is still considered as good clinical practice.

Serum progesterone (P4) measurement during the ovarian hyperstimulation is gaining importance. Progesterone levels could have an effect on the pregnancy rate. Sighn et al. (2015) investigated the effect of progesterone and progesterone/estradiol levels and found a negative association be- tween pregnancy rate (PR) and serum P4 and P4/ E2 levels with no effect on fertilization and cleavage rate. That is why we undertook the present in- vestigation, because until now patients choosing for home monitoring were not followed using serum E2 measurements and it would create a patient-friendly approach if home saliva measurements could be used replacing serum determinations necessitating repeated phlebotomies.

Saliva has been shown to be a stress-free, non- invasive and practical matrix for measurement of hormones such as cortisol (C) and testosterone (T) (Estrada-Y –Martin et al., 2011, Fiers et al., 2014). In serum only a small free fraction functions as the active hormone and this fraction can be measured by reference methods such as equilibrium dialysis (ED) coupled to liquid chromatography with tandem mass spectrometry (LC-MS/MS) (Kirchhoff et al., 2011). In saliva hormones are largely present in their free, unbound form, even if there has been shown to be some binding to salivary proteins for free T in women (Fiers et al., 2012). For E2 a similar mechanism based on the Law of Mass Action is suggested. In blood E2 is strongly bound to sex hormone binding globuline (SHBG) and weakly to albumin. It is assumed that in women about 1% of E2 is actually present as free hormone in blood and calculations have been suggested for E2 to estimate this free fraction (Rosner et al., 2015). In the past it has not been investigated whether salivary E2 could be a good surrogate marker for serum E2 by LC-MS/MS nor how direct measurement of free E2 in serum by ED-LC-MS/MS correlates to salivary E2. The main reasons therefore were that the serum concentrations of E2 in physiological cycles are low making measurements hazardous in their technicity and interpretation. The clinical question is if E2 in saliva could be used as a low cost and stress-free potential surrogate marker for predicting OHSS in woman undergoing ART.

## Aim of study

We wanted to determine whether there is a correlation between saliva- and serum concentrations of E2, in women undergoing ovarian hyperstimulation with gonadotropins for in IVF treatment. The goal is to simplify the follow up of an IVF/ICSI treatment. In order to make home-monitoring possible and in-hospital follow up easier, we would need to find a simple way to test E2. If a good correlation between serum and saliva measurements exists, it would make sense to try and develop a POC (Point Of Care test) for E2.

## Materials and Methods

### Study design

This study was approved by the ethical committee of the University hospital Ghent (EC B670201421937). Written consent was obtained from all patients who cooperated in the study.

A prospective interventional academic monocentric investigation was performed at the Centre for Reproductive Medicine of the University Hospital of Ghent between November 2014 and August 2015. Thirty-one patients, treated with gonadotropins for ovarian hyperstimulation for IVF/ICSI treatment, were included between November 2014 and August 2015. Patients were selected at random and had to fulfil the following inclusion criteria: < 41 years of age, any rank of IVF/ICSI cycle, serum anti -Müllerian hormone ≥ 1 μg/L and the complete treatment had to be conducted in the UGent academic hospital. Measurements took place in the laboratory of hormonology of the University hospital of Ghent. Serial serum and saliva samples were collected from the patients.

At every hospital visit, from the start of treatment until the first pregnancy test, measurements of serum E2, salivary E2 and a equilibrium dialysis on a subset of the serum samples were performed. The equilibrium dialysis was done, since E2 in the serum is largely bound to SHBG, and also to albumin. Equilibrium dialysis for E2 in the serum was performed, to make sure that free concentration in the serum correlates with the total concentration.

Blood samples were collected after phlebotomy at the fertility centre of the University hospital of Ghent. Saliva sampling took place by ‘passive drooling’, i.e. collecting saliva by letting it run into a polypropylene test tube (4 mL) by a straw. Instructions for saliva sampling were given in written to the patients.

The technical details of the laboratory techniques used are described by Fiers et al (Fiers et al., 2016). Statistical analysis was performed by the Department of Statistics of the University Hospital of Ghent. The linear mixed-effects models (MIXED) procedure in SPSS was used for statistical analysis.

## Patient characteristics

Patients’ age ranged from 23 - 38 years, BMI from 18,9 - 30,4 kg/m2. Twenty of the thirty-one patients were treated with antagonist plus human meno- pausal gonadotropins, nine with agonist and human menoposal gonadotropins and one with agonist and recombinant gonadotropin. There was a mix of all infertility causes (andrological, gynaecologic, idiopathic) . All patients were treated in the University of Ghent hospital and all TVUS and blood and saliva samples were done there. Treatment follow up comprised between four to nine visits.

## Results

Table I shows the results for serum and salivary E2 concentrations on a subset (random) of the samples equilibrium dialysis (serum ED-E2) was performed in order to measure free E2 in the serum.

**Table I:** — Serum and salivary E2 concentrations and equilibrium dialy- sis (serum ED-E2). Data distribution (subset) of obtained E2 values in serum, saliva, and serum post equilibrium dialysis in pg/ml (pmol/L)

	Serum total E2	Serum ED-E2	Saliva E2
N	178	26	177
Median	669 (2456)	7.2 (26.4)	4.68 (17.2)
5th percentile	7.97 (29.2)	0.5 (1.84)	0.3 (1.10)
95th percentile	2629 (9651)	18.0 (66.1)	18.7 (68.6)

Serum and salivary E2 concentrations and equilibrium dialysis (serum ED-E2). Data distribution (subset) of obtained E2 values in serum, saliva, and serum post equilibrium dialysis in pg/ml (pmol/L)

Serum values before and after equilibrium dialysis concur with the literature concerning the estimated 1% free fraction of E2 in the serum (Rosner et al., 2015). The 5-95th percentile range for salivary E2 is almost identical with the serum E2 after equilibrium dialysis.

**Figure 1:**
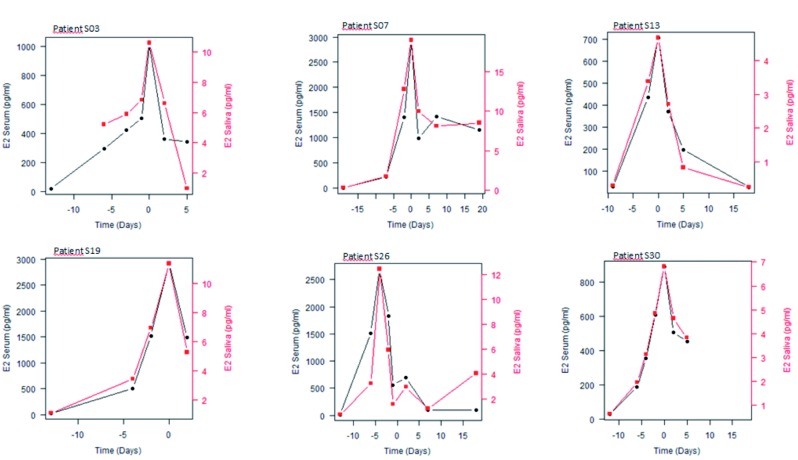
— Variation of salivary and serum E2 in individual patients. Six exemplary graphical overlay line plots.

First we looked at the variation of salivary and serum E2 in individual patients in order to see the ‘natural’ variation during stimulation in serum and salivary E2. An overlay plot for serum versus saliva per patient is represented in a graphical line plot, with different scales for salivary and serum E2, in order to show a possible parallelism between variations of salivary and serum E2 concentrations. Time is expressed per day in relation to the ovulation trigger (human chorionic gonadotropin = hCG = Pregnyl®). Figure 1 shows six exemplary graphical overlay line plots.

For the overall mean trend and evolution in time of serum- E2, and salivary-E2, an explorative analysis was done. Five measurement points were taken into account in each cycle: at the start, at day LH minus 3 days (LH-3), at the day of triggering of ovulation (LH), the day of ovum pick up (= OPU), i.e. two days after triggering (= LH + 2) and at the day of embryo transfer performed on day five after ovum pick up ( LH+7).

We used neighbouring measurements as single imputation for missing measurements on the set of measure points in the stimulation cycle (start, LH-3, LH, LH + 2 and LH+7). The following imputations are used: LH- 1 if LH is missing, LH+5 if LH+ 7 is missing, LH-4 if LH-3 is missing and LH-2 if as well LH-4 and LH-3 were missing. This is acceptable since we are examining a trend in the cycle, and we wanted to see whether the course of E2 changes during hyperstimulation in a comparable way both in serum and saliva.

**Figure 2:**
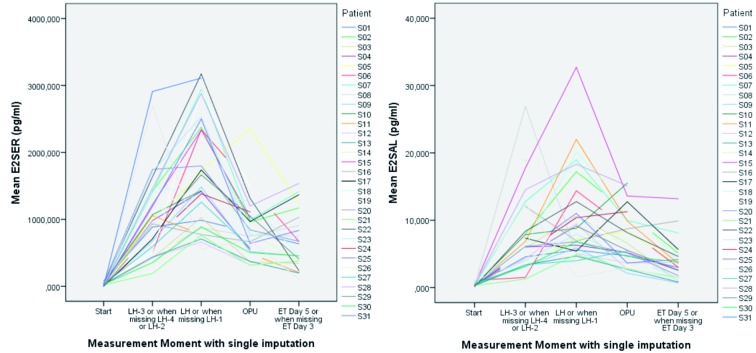
— Overall mean trend and evolution in time of serum- E2 and salivary-E2. Left graphic shows serum E2 and right graphic salivary E2 for each patient at the different measure points. Notice the comparable course for E2 in saliva and serum.

Based on this data set further comparative analysis was performed. When we compare the graphics for serum E2 and salivary E2, with this single imputation we see a comparable course for E2, as shown in figure 2.

Table II shows a very low Pearson coefficient (R) for E2 serum versus salivary (- 0,046) at the moment of start, due to low concentrations. During the rest of the cycle we see good correlations for E2 serum and E2 salivary, with high Pearson coefficients (R). We also see a good Pearson correlation between E2 dialysis and E2 serum.

**Table II:** 

		E2 SAL (pg/mL)	E2 SER (pg/mL)	E2 DIAL
**Start of treatment**				
E2 SAL (pg/mL)	Pearson correlation	1	-0,046	.a
E2 SER (pg/mL)	Pearson correlation	-0,046	1	1,000**
E2 DIAL	Pearson correlation	.a	1,000**	1
			
**LH-3 or when missing LH-4 or LH-2**				
E2 SAL (pg/mL)	Pearson correlation	1	,692**	0,199
E2 SER (pg/mL)	Pearson correlation	,692**	1	,963**
E2 DIAL	Pearson correlation	0,199	,963**	1
			
**LH or when missing LH-1**				
E2 SAL (pg/mL)	Pearson correlation	1	,628**	,738*
E2 SER (pg/mL)	Pearson correlation	,628**	1	,924**
E2 DIAL	Pearson correlation	,738*	,924**	1
			
**OPU**				
E2 SAL (pg/mL)	Pearson correlation	1	,491*	0,942
E2 SER (pg/mL)	Pearson correlation	,491*	1	0,564
E2 DIAL	Pearson correlation	0,942	0,564	1
			
**ET Day 5 or when missing ET Day 3**				
E2 SAL (pg/mL)	Pearson correlation	1	,647**	1,000**
E2 SER (pg/mL)	Pearson correlation	,647**	1	1,000**
E2 DIAL	Pearson correlation	1,000**	1,000**	1
			

Pearson coefficient (R) for E2 serum versus salivary E2 and Pearson coefficients ( R) for E2 dialysis and E2 serum at different time points in the stimulation

A reduction in Level 1 variance of E2 serum was performed (R2) for E2 serum in function of E2 saliva. An analysis based on the random intercept model was performed for every patient. The linear relation between the mean E2 serum and E2 saliva was derived from these measurements. We used all measurements for this analysis.

**Figure 3:**
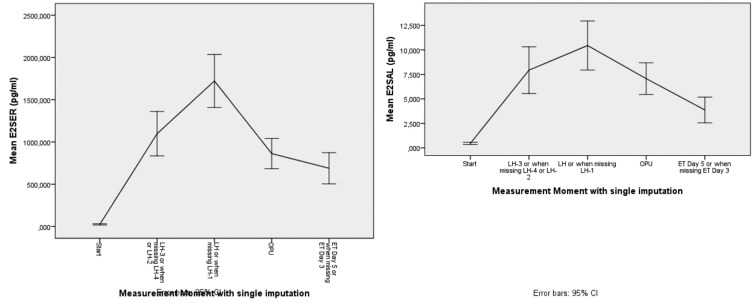
— Mean course of E2 in serum and saliva

An overall reduction in level 1 variance was calculated to be 0,742 (Fig. 4a). This shows a high correlation between E2 serum and E2 saliva, meaning that 74,2% of the level in E2-serum variance is accounted by E2 saliva at level 1. The results for the serum versus equilibrium dialysis we also have a high reduction in level 1 variance.

**Figure 4a:**
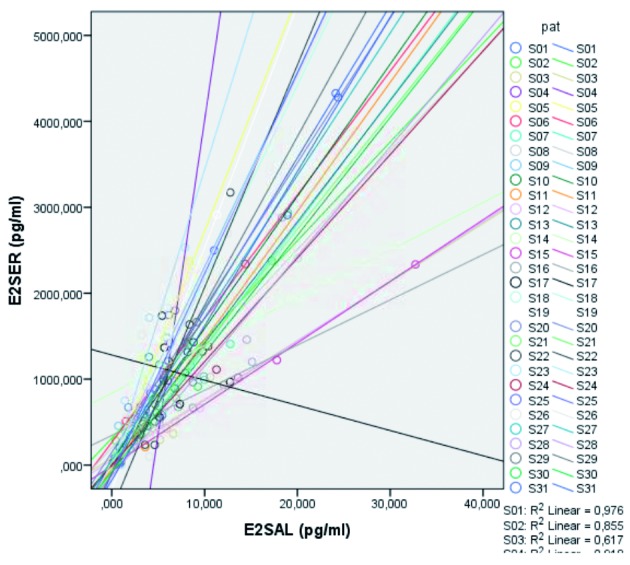
— Reduction in Level 1 variance of E2 serum (R2) for E2 serum in function of E2 saliva. The graph shows high correlation between E2 serum and E2 saliva.

The graph in Figure 4b shows the linear relation between E2 after dialysis and E2 in serum for every patient. An overall reduction in level 1 variance was calculated to be 0,854. This shows a high correlation between E2 serum and E2 after dialysis. Please note that there are only a limited number of occasions where both E2 serum and E2 dialysis measurements were present. 85.4% of the level-1 variance in E2 serum is accounted by E2 dialysis at level 1.

**Figure 4b:**
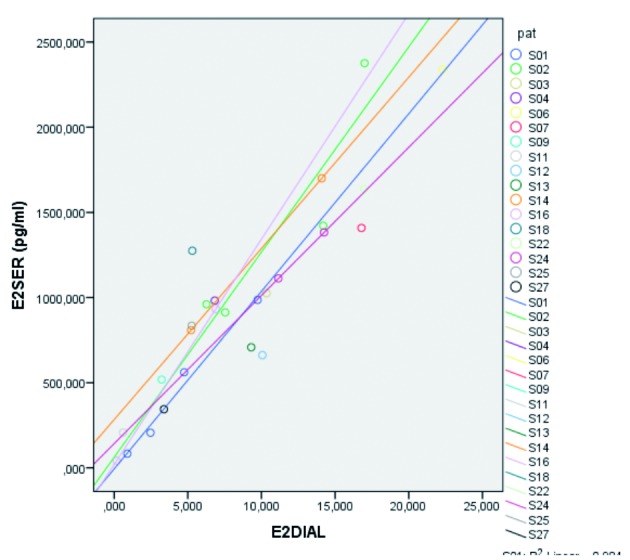
— Reduction in Level 1 variance. Linear relation between E2 after dialysis and E2 in serum for every patient. The graph shows high correlation between E2 serum and E2 after dialysis.

## Discussion

For the follow-up of ovarian hyperstimulation prior to ART, TVUS is golden standard and serum sampling for E2 is considered as good clinical practice. Follow up is time consuming and has downsides for patients and care providers. Alternatives for TVUS would be welcome.

Saliva is an attractive practical and stress-free alternative to blood, which can be collected by the patients themselves (at home or in the hospital). Since food intake can influence the salivary concentrations of estradiol, patients need to be informed and instructed, to avoid false results. For example it is known that use of chewing gum can influence concentrations in salivary estradiol.

First of all, a correlation between saliva and serum values of E2 needed to be shown, to assess whether saliva has the potential for the development of a point of care test in the future, thereby making its measurement more easy and simplifying the follow-up for ART patients. The test should be able to make a distinction between women at risk for OHSS and women less at risk, identifying candidates for coasting and/or an all-freeze strategy. Its results should ideally be evaluated by a physician. In the past E2 and P4 have shown to be bad candidates for salivary testing, presumably because of their low concentrations during the normal menstrual cycle. In case of ovarian hyperstimulation however, concentrations tend to rise 50-100 fold higher. In this supraphysiological setting the development of a point of care test would make sense and is more likely to be feasible.

Our investigation shows that, with for moment of measurement in time, there is good correlation between serum and salivary E2 in every patient. We conclude that serum E2 correlates well with salivary E2 (R2 = 0,74) and that serum E2 correlates well with equilibrium dialysis E2 (R2 = 0,85). Evidence in this pilot study is provided to support the hypothesis that salivary E2 could be used as an indicator for serum E2.

Similar dynamics for saliva and serum E2 support the hypothesis that salivary E2 might indeed be considered as a surrogate marker for serum E2 in ART patients. Salivary estradiol testing is not yet commercially available although the technology is being evaluated in on-going trials.

We didn’t search for correlation between serum and salivary P4. For the future development of a POC test P4 is not a priority, since it is more important as an optimizing approach for implantation (Singh et al., 2015]). The care provider can still decide to examine P4 serum value once during ART cycle, for example just before hCG administration.

## Conclusion

A good correlation between serum and salivary estradiol is found. Equilibrium dialysis shows a good correlation with salivary E2. Saliva can be a good surrogate for free E2, in women undergoing ovarian hyperstimulation. This creates the opportunity to develop a POC for E2 measurement, with the purpose to simplify screening for an OHSS risk.
